# “I RUN CLEAN Project”—An Innovative and Self-Sustainable Approach to Develop Clean Sport Behaviours in Grassroots Athletes

**DOI:** 10.3390/ejihpe13110178

**Published:** 2023-11-08

**Authors:** Roberto Codella, Fabio Lucidi, Fabio Alivernini, Tommaso Palombi, Bill Glad, Jean Gracia, Daniel Gotti, Antonio La Torre, Andrea Chirico

**Affiliations:** 1 Department of Biomedical Sciences for Health, Università degli Studi di Milano, 20133 Milan, Italy; roberto.codella@unimi.it (R.C.); daniel.gotti@unimi.it (D.G.); antonio.latorre@unimi.it (A.L.T.); 2 Department of Endocrinology, Nutrition and Metabolic Diseases, IRCCS MultiMedica, 20138 Milan, Italy; 3 Department of Social and Developmental Psychology, Sapienza University of Rome, 00185 Rome, Italy; fabio.lucidi@uniroma1.it (F.L.); fabio.alivernini@uniroma1.it (F.A.); tommaso.palombi@uniroma1.it (T.P.); 4 Agency for the Development of Athletics in Europe, 75013 Paris, France; bill.glad@euroathledev.eu (B.G.); jean.gracia@european-athletics.org (J.G.)

**Keywords:** anti-doping, athlete behaviours, educational programme

## Abstract

The phenomenon of doping is a public health issue that poses threats to sport and society. In recent decades, the emphasis on efforts to address the issue and reduce the incidence of doping by young people in sport has shifted from deterrence through testing and punishment to the promotion of clean sport behaviours through values-based education. The “I Run Clean project” sought to develop new and effective tools targeting grassroots athletes and those around them (coaches, medical support personnel, sport leaders, parents). These included sport-specific e-learning and in-person peer-to-peer workshops led by trained volunteer ambassadors. The aim of all “I Run Clean” measures is to go beyond the warnings and provision of factual information about early anti-doping campaigns to a more holistic educational approach that focuses participants on their personal and sport-related values in order to encourage good decision-making and resistance to doping-related behaviours. This study evaluates the efficacy of the peer-to-peer workshops and their impact on selected psycho-social variables. The collaboration of the volunteer ambassadors is shown to effectively transmit the desired reasoning, reduce doping risk factors and enhance protective factors.

## 1. Introduction

The phenomenon of doping is a public health issue that poses threats to sport and society, both in Europe and worldwide. The use of drugs and other potentially harmful substances to enhance athletic performance is a persistent issue in both professional and amateur sports, but evidence shows that it is not restricted to a small number of elite performers. There are concerns that more and more young people in grassroots and recreational sport are using substances prohibited by the World Anti-Doping Code [1,2,3]. From a broader perspective, there is also evidence of increasing use of “performance and image enhancing drugs” or PIEDs—substances (including food supplements) used to promote muscle growth, reduce body fat, increase energy and enhance concentration—by high school students and non-athlete adolescents, often without medical supervision and in amounts that greatly exceed recommended therapeutic doses [4,5,6,7,8,9].

Initial efforts to combat doping in sport focused primarily on punishment—identifying users through increasingly rigorous testing and imposing bans from competition. However, historical evidence shows that these measures alone have been insufficient. These theoretically deterrent measures have been supplemented with a number of information programmes and prevention campaigns conducted by government agencies and sporting bodies at both the national and international levels [2,10,11]. Although they generally feature strong, well-prepared content emphasising the risks to health, the rules and doping control procedures, their impact on behaviours is not always clear. Indeed, the findings of the European Commission’s Study on Doping Prevention [12] include considerations that create instability in the belief that such measures have had a tangible effect, either with elite performers or other young people.

Over the past decade, researchers have explored doping behaviour from a psychological standpoint [9,13,14]. It has been viewed as a goal-directed behaviour, with the “intention to dope” playing a crucial role. From this starting point, there has been increasing emphasis on the role of values and other aspects of morality. In the context of predictive factors, strong positive personal values have come to be considered protective. This approach highlights the importance of fostering compliance with ethical principles through education [15,16,17].

Article 18 of the first version of the World Anti-Doping Code (2003) [18] simply stated that anti-doping education programmes “should promote the spirit of sport in order to establish an anti-doping environment which influences behaviour among *Participants*” and called on *Athlete Support Personnel* to “educate and counsel *Athletes* regarding anti-doping policies and rules”. The 2009 updated code [19] moved beyond recommending the provision of policy, rules and basic information with the addition of “Prevention programmes should be values-based”. In 2014, the World Anti-Doping Agency (WADA) launched the online Athlete Learning Program about Health and Anti-Doping (ALPHA) [20] targeting athletes from all sports around the world, which included an approximately 10 min-long unit on “Ethical reasons not to dope”. However, it was not until the WADA staged a Values-Based Education Conference in 2015 and published its International Standard for Education in 2021 [21] that official international guidelines for implementing values-based anti-doping education were available to other sports organizations.

During the period in which the concept of values-based anti-doping education was still developing, *European Athletics*, the continental governing body for the sport of track and field athletics, created its own highly interactive e-learning platform called “I Run Clean”, which was launched in 2017 [22]. I Run Clean was (and remains) available in 26 languages, free to use and open to anyone, although the imagery and examples clearly targeted participants in the sport of athletics. A defining feature of I Run Clean is the initial module (of eight), entitled Making Good Decisions, which is intended to raise awareness of the user’s personal values and how these inform his/her decisions, including whether or not to dope. Unlike WADA’s voluntary ALPHA, or the Anti-Doping e-Learning (ADeL) platform that superseded it in 2018 [23], European Athletics made the completion of I Run Clean a mandatory entry requirement for top athletes to participate in any of its championship events. The platform has attracted in excess of 49,000 users as of September 2023, more than 41,000 of whom have completed the entire programme.

The initial success of the platform led to a 2020 follow-on project [24], co-funded by the European Union and delivered by a consortium that included six national athletics federations representing a cross section of European Athletics’ 51 members, which is the subject of the current study. This novel project aimed to impact the culture of the sport by leveraging the message that completing I Run Clean is an accepted part of being a responsible elite athlete and targeting a wider set of groups—grassroots athletes, students, coaches, medical support personnel, leaders and parents—with new group-specific modules using the same values-based approach. In addition, the project plan included complementing the top–down approach of the I Run Clean e-learning platform with workshops adapted for each group to promote peer-to-peer discussion of values, following the premise that moral attitudes develop most sensitively in person-to-person relationships. The thinking was that the unique double intervention (e-learning and workshops) would provide greater educational value to the various target groups and credibly link the international and local levels of the sport in a common effort to control doping.

The specific objectives for the 2020 I Run Clean follow-on project included:

- To develop anti-doping workshops for settings such as schools, sport clubs, training camps and competitions.
- To develop a process to prepare young volunteer ambassadors to deliver the anti-doping workshops.

The use of volunteer ambassadors was intended to maximise the number of I Run Clean workshops that could be delivered in each country and help ensure the sustainability of the overall programme by minimising costs. Although they would be provided with relevant information on many aspects of the fight against doping, these ambassadors were not expected to become experts on the topic or, importantly, to teach the workshop participants, many of whom were likely to be older and more experienced. Rather, it was intended that they would be able to effectively facilitate an exchange of views about values and ethics and promote positive values within a semi-flexible workshop structure prepared by the central project team, thereby allowing the workshop participants to learn from their peers. The ability to competently manage discussions among diverse target groups was, therefore, of paramount importance. The development of these skills and related experience were offered as career-enhancing benefits to the volunteers participating in the project.

The purpose of the current study, therefore, was to start building an understanding of how I Run Clean and similar programmes can affect attitudes by evaluating the quality and impact of key elements of the peer-to-peer workshops added through the follow-on project.

The specific research areas concerned are (1) the quality of the preparation of the young volunteer ambassadors tasked with delivering the workshops; and (2) the overall impact of the first set of target-group specific workshops on participants with regards to the key variables of self-regulatory efficacy, moral disengagement, moral identity and attitudes towards doping.

## 2. Materials and Methods

### 2.1. Project Activities

The I Run Clean follow-on project was conducted between 1 January 2020 and 31 March 2023. The work streams included the design and production of the new e-learning modules for coaches and other target groups, as well as drafting the structure and contents for the athletes’ and coaches’ workshops, which were carried out by the central project team. Of particular interest for the present study were the recruitment and preparation of the volunteer ambassadors to deliver the workshops, as well as the organization and staging of the workshops by the participating federations, which involved both the central project team and participating federations. Data for the evaluation tools were collected following the main preparation of the volunteers and throughout the workshop delivery period.

### 2.2. Ambassador Recruitment and Preparation

*Recruitment*: Ambassadors (*n* = 39) were recruited in early 2021 through the participating federations’ networks, including contacts with athletics clubs and traditional media channels, particularly their websites. These efforts were based on the following ideal ambassador profile:

- Aged between 20 and 30;
- Interested in sports/athletics (but not currently an active athlete);
- Values fair play and integrity of sport;
- Fluent in English;
- Possesses teaching skills (for example: teachers, trainee teachers and young coaches);
- Outgoing, proactive and self-confident;
- A team worker;
- Available for all required tasks in 2021 and 2022;
- Motivated to work voluntarily.

*Preparation*: The aims of the central project team-led ambassador preparation process were:

- To build a sense of team and shared commitment to the success of the I Run Clean programme among the ambassador cohort.
- To assist the ambassadors in developing the background knowledge and facilitation skills required to effectively conduct the Run Clean workshops.
- To prepare the ambassadors to communicate about and promote the I Run Clean programme (workshops, e-learning platform, on-going engagement).
- To lay the groundwork for ambassadors to be able to prepare the next generation of ambassadors in their countries.

The process, which took place throughout the summer of 2021, included (1) four cycles of reading assignments and optional online discussion groups to provide background information, address questions and allow the ambassadors to become acquainted with their colleagues and the central project team and (2) a three-day training camp, which took place in Berlin, Germany, 1–3 October 2021. The contents of the training camp included:

- Creating a good workshop atmosphere;
- Leading workshop discussions (including practical experience facilitating various sized groups of colleagues in selected elements from the workshop structure);
- Troubleshooting;
- Communications and the promotion of the I Run Clean programme.

Post-training camp support was provided by the central project team through an online ambassador newsletter that included details of the workshops delivered, updates on the workshop structure, PowerPoint presentations and other information of interest.

### 2.3. Ambassador Preparation Evaluation Tool

To evaluate the effectiveness of ambassadors’ preparation, an oral presentation self-efficacy questionnaire was used. The aim was to elicit data from ambassadors about their oral presentation self-confidence as a proxy for the self-confidence required to lead workshops for diverse groups of participants.

Wattananan and Tepsuriwong [25] formatted the questionnaire items in the form of a 5-point Likert scale ranging from strongly agree (5) to strongly disagree (1). The scale comprised 20 items with two subscales for measuring the different aspects of oral presentation self-efficacy beliefs. The subscales include general self-efficacy beliefs and an affective states component (e.g., anxiety). The internal consistency of the tool at the scale level was 0.87 in Cronbach alpha. The subscales had consistency values of 0.88 for the affective states subscale. Therefore, the tool was reliable and suitable for measuring the relevant construct.

The data were collected online in October 2021.

### 2.4. Workshops

*Structure and contents*: The basic outlines for the workshops were developed by the central project team and documented, normally with drawings, bullet points and short phrases in English on PowerPoint. It was anticipated that the workshops would follow the given outlines, but the ambassadors would have some flexibility to develop points or lines of discussion based on the interests of the participants and the flow of the conversation.

Ambassadors in each of the participating countries translated the slides to their national languages, and the final product was checked by coordinators from the respective national athletics federations.

*Organisation*: The pilot workshops covered in the current study were promoted and staged by the federations—at athlete training camps, in clubs and sometimes in the federation headquarters—and delivered by the ambassadors (2–3 per workshop). The recommended group size to allow an optimal amount of participant input and discussion was 15–20.

### 2.5. Psychosocial Variables and Evaluation Tools

To evaluate the effectiveness of the workshops, data were collected via online surveys pre- and post-intervention for the following psychosocial variables using the tools described below.

*Self-regulatory efficacy*: According to Social Cognitive Theory (SCT) [26,27], “perceived self-efficacy” refers to individuals’ beliefs about their ability to achieve goals and overcome challenges. In the context of doping, where social pressures and the influence of peers may be significant, one’s own belief in their ability to resist such pressures is crucial. This is known as “doping-specific self-regulatory efficacy”, which involves resisting social pressure and avoiding situations where doping is likely to occur. Several studies have demonstrated that self-regulatory efficacy toward doping is a strong predictor of doping intentions and self-reported doping use [13,28].

A sport-specific version of the doping self-regulatory efficacy scale has been used to measure the perceived ability to resist doping [14]. This version has been used and validated in several research studies [10]. Athletes are asked to indicate their confidence in their ability to avoid using banned substances to improve performance in sport in seven situations (e.g., “When pressured to do so by others”) using a 7-point scale anchored by 1 (not at all confident) and 7 (completely confident). The scale has shown excellent internal consistency (α = 0.96–0.97; [8,9]). The scale has been validated and used in the Greek, Italian and French languages [13,14].

*Moral disengagement*: Moral disengagement (MD) is a key concept within Social Cognitive Theory (SCT) [17]. It can be defined as a self-regulatory process that allows individuals to engage in doping while still perceiving their actions as morally acceptable. MD mechanisms can be categorised based on their focus: relabelling doping as a “supplement” through the use of euphemistic language, distorting or downplaying the negative effects of doping by claiming it does not harm anyone and shifting responsibility by attributing the decision to external factors such as a coach’s request. These mechanisms serve to justify and rationalise doping behaviour. Both cross-sectional and subjective examinations have reliably reported the positive relationship of MD with doping conduct in competitors across various ages and performance levels [29,30].

Dispositional doping moral disengagement has been measured using the 6-item scale developed by Lucidi et al. [14] to assess moral disengagement toward doping. Participants were asked to rate their level of agreement with statements on a 7-point Likert type scale, anchored by 1 (strongly disagree) to 7 (strongly agree). Each item assesses one of the six mechanisms of moral disengagement used to justify doping in previous research. Psychometric support for the scale has been provided with an alpha coefficient of 0.84 [26]. The scale has been validated in English, Italian and French [10,31].

*Moral identity*: Moral identity refers to the cognition people hold when thinking about their moral character and their desire to be a moral person. Moral identity has been reported [10,29,30] as one of the strongest predictors of moral disengagement after gender. In other words, the desire to be a person with positive values negatively predicts risk factors for doping, such as MD.

Moral identity has been evaluated using the 5-item internalisation subscale of the moral identity scale [30], adapted to sports contexts [27]. This subscale taps the degree to which moral traits are central to individuals’ self-concept [30]. Participants were presented with nine traits (e.g., caring, fair, kind, helpful) validated as necessary characteristics of a moral person [30] and were asked to respond to statements concerning these traits (e.g., “It would make me feel good to be a person who has these characteristics”). Responses were put on a 7-point scale, anchored by 1 (strongly disagree) and 7 (strongly agree). Reed and Aquino [30] have provided evidence for the reliability (0.83) of this subscale, as well as Kavussanu et al., who reported good reliability, with an alpha coefficient of 0.82 [27].

*Attitudes toward doping*: According to the Theory of Planned Behaviour (TPB), which is a precursor to Social Cognitive Theory (SCT), “attitude” refers to individuals’ positive or negative evaluations of a particular behaviour. In the context of doping, attitude reflects one’s positive or negative assessment of its use. Research based on the TPB has consistently shown that attitudes toward doping are effective predictors of doping intentions and behaviour. Numerous studies have confirmed the significant impact of attitude on doping susceptibility and behaviour among competitive athletes, as well as non-competitive athletes in various populations and settings [11,32,33].

Attitudes toward performance-enhancing substances have been evaluated through a short version of the Performance Enhancement Attitude Scale (PEAS). The 5-item short version comprises a one-dimensional self-report factor measuring general attitudes toward doping. The final response format is a 6-point Likert-type scale, with points anchored as strongly disagree (1), disagree (2), slightly disagree (3), slightly agree (4), agree (5) and strongly agree (6). No neutral response option is offered, and all the items are scored in the same direction. Thus, the overall PEAS score ranges from 17 to 102. The reliability of the scale in different studies ranged between 0.71 and 0.91 (across various samples), providing good evidence of the scale’s reliability. Construct validity has also been thoroughly evaluated [34].

The time needed to complete the surveys was approximately 10 min. These were administered between November 2022 and February 2023.

### 2.6. Data Analysis

Descriptive analyses were used on the sample characteristics (i.e., sociodemographics). The Gaussian distribution of the data was ascertained using skewness and kurtosis indexes, both within the conventional cut-off [35]. The main analyses relied on *t*-tests for paired samples and a series of one-way repeated measures ANOVA to test the possible intervention effects over time on athletes’ and coaches’ moral disengagement, positive attitudes toward doping, moral identity and self-efficacy. Statistical analyses were performed using the Jamovi software v. 3.2 (The jamovi project, 2023), employing statistical significance at α = 0.05.

### 2.7. Ethics

The Department of Psychology of Development and Socialization Processes Ethical Committee of the “Sapienza” University of Rome approved the study. All participants were informed of the general purpose of the study and their rights to anonymity. The researchers obtained written informed consent from each participant. Collected data were coded and processed anonymously.

## 3. Results

### 3.1. Ambassador Preparation Effectiveness

Thirty-two ambassadors (M age = 25.9 yrs; SD age = 2.66; 65% female) of the thirty-nine that took part in the Berlin training camp completed the ambassador survey questionnaire.

The results of the paired sample *t*-test showed a significant improvement in the public speaking self-efficacy of the participants and a reduction in the affective states (*p* < 0.01) that could diminish performance when speaking at a public event such as a workshop. The results are reported in Table 1.

### 3.2. Effectiveness of the Workshops

A total of 616 workshop participants completed the pre- and post-workshop questionnaires. This group comprised 378 athletes (52.8% Male; M age = 17.3 yrs; SD age = 3.2) and 238 coaches (71% Male; M age = 48.8; SD age = 15.8). The breakdown of nationalities within the entire sample is as follows: 22.6% Bulgarian, 9.5% Estonian, 21.8% French, 6.7% German and 39.3% Italian.

#### 3.2.1. Self-Regulatory Efficacy

**Figure 1:**
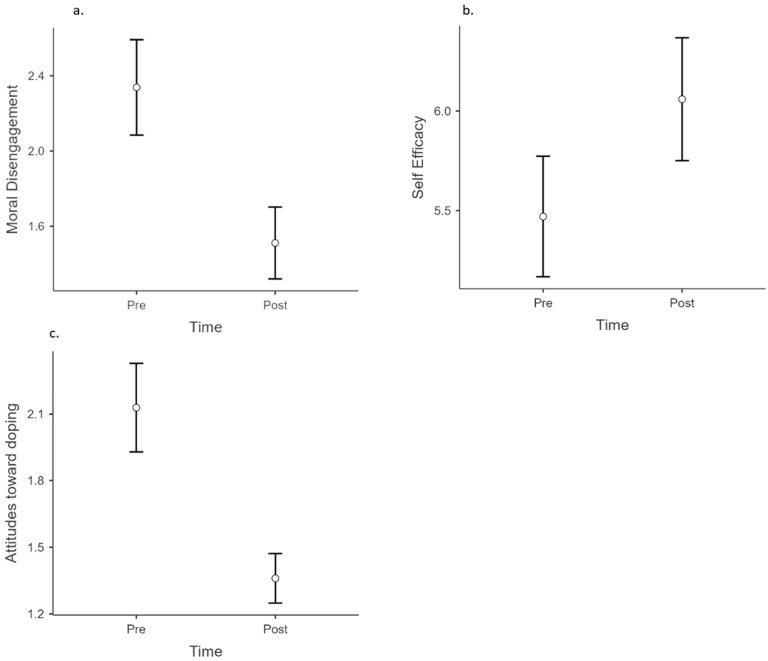
Impact of the workshops on coaches’ psychosocial variables. Scoring is reported on the Y axis (0–5 intensity scale). (**a**) Moral disengagement output. (**b**) Self-efficacy output. (**c**) Attitudes towards doping output.

The results of the ANOVA showed no significant effect of the intervention on the athletes’ perceived self-efficacy (F (1, 184) = 1.17; *p* = 0.281; partial eta square = 0.006) and a significant effect on coaches’ perceived self-efficacy (F (1, 127) = 14.5; *p* < 0.001; partial eta square = 0.103). To visualise this effect, Figure 1b shows the coaches’ self-efficacy mean scores across experimental timepoints. The self-regulatory efficacy scale showed good reliability, with an alpha coefficient of 0.97.

#### 3.2.2. Moral Disengagement

**Figure 2:**
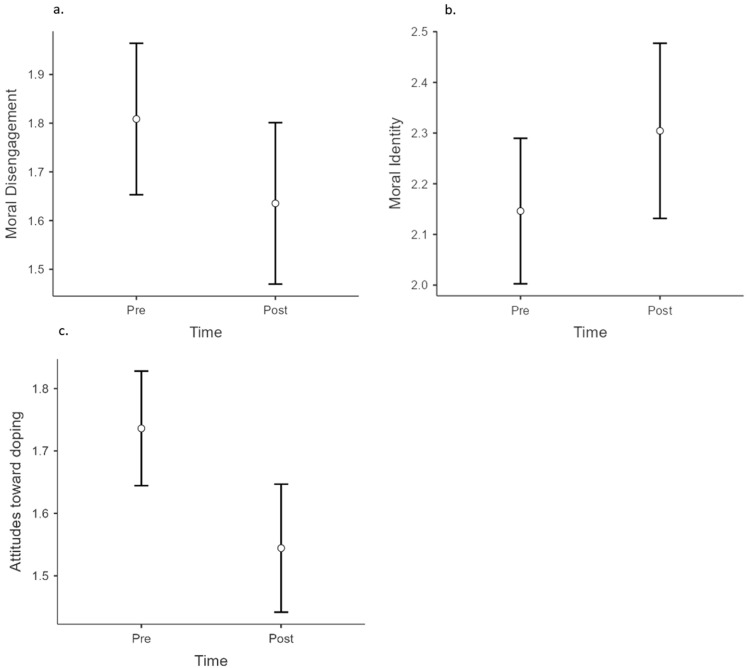
Impact of the workshops on athletes’ psychosocial variables. Scoring is reported on the Y axis (0–5 intensity scale). (**a**) Moral disengagement output. (**b**) Moral identity output. (**c**) Attitudes towards doping output.

The results of the ANOVA showed that there was a significant reduction in both the athletes’ moral disengagement (F (1, 201) = 4.45; *p* < 0.05; partial eta square = 0.022) and the coaches’ moral disengagement (F (1, 132) = 45.4; *p* < 0.001; partial eta square = 0.198). To visualise these effects, Figure 1a and Figure 2a show the coaches’ and the athletes’ moral disengagement mean scores across experimental timepoints. The moral disengagement scale showed good reliability, with an alpha coefficient of 0.86.

#### 3.2.3. Moral Identity

The results of the ANOVA showed a significant improvement in the athletes’ moral identity (F (1, 181) = 6.35; *p* < 0.05; partial eta square = 0.034). To visualise this effect, Figure 2b shows the athletes’ moral identity mean scores across experimental timepoints. However, the ANOVA showed no significant intervention effect on coaches’ moral identity (F (1, 126) = 1.60; *p* = 0.209; partial eta square = 0.013). The moral identity scale showed good reliability, with an alpha coefficient of 0.81.

#### 3.2.4. Attitudes toward Doping

The results of the ANOVA showed that there was a significant reduction in both the athletes’ attitudes toward doping (F (1, 191) = 21.2; *p* < 0.001; partial eta square = 0.100) and the coaches’ attitudes toward doping (F (1, 129) = 62.2; *p* < 0.001; partial eta square = 0.325). To visualise these effects, Figure 1c and Figure 2c show the coaches’ and athletes’ attitudes toward doping mean scores across experimental timepoints. The scale showed good reliability, with an alpha coefficient of 0.85.

## 4. Discussion

This study aimed to evaluate the impact of anti-doping education workshops targeting grassroots athletes and coaches. Another primary endpoint of this research was the quality of the preparation of the young volunteer ambassadors who deliver the workshops. It provided a positive assessment of the workshop and contents. It also confirmed the delivery capabilities of the ambassadors.

The bottom-line measure of the success of any anti-doping education intervention is, of course, the impact on relevant psychosocial variables in the participants. The study clearly demonstrated the short-term effectiveness of the workshops. The results of the surveys administered in the two target groups studied (athletes and coaches) showed reduced risk factors (attitudes toward doping (Figure 1c and Figure 2c); moral disengagement (Figure 1a and Figure 2a)) and enhanced protective factors (self-regulatory efficacy (Figure 1b); moral identity (Figure 2b)). The results are in line with other anti-doping interventions aimed at influencing attitudes toward doping [2], moral disengagement [36], as well as enhanced self-regulatory efficacy [37] and moral identity [15].

The presented results show the particular efficacy of the interventions focused on coaches, addressing the important need for coaches to provide positive role models and foster a clean sport environment. In fact, past evidence has shown that, besides peers, coaches are an important source of influence for athletes [38]. Athletes and coaches have reported that having positive role models in their immediate environment can be critical. Role models can affect decisions toward health-related behaviours [39]. At the same time, the environment is relevant for the decision-making process [40]. Importantly, this influence can be either in favour or against doping, affecting both athletes and coaches. Hence, anti-doping education should aim to change athletes’ and coaches’ beliefs towards doping and promote positive role models to foster a clean sport environment [38]. Nevertheless, the impact shown on the athletes was somewhat less; a possible explanation is a putative distortion effect originating from the different sample sizes per group/country.

With regard to the ambassadors, the study indicates that the preparation process used can enhance the capability of selected young ambassadors to lead workshops. The results extrapolated from the oral presentation self-efficacy questionnaire administered to the ambassadors showed that the training process substantially improved their confidence in public speaking (effect size: 1.254, pre-post evaluation, Table 1), which is important for their ability to lead discussions about any topic, including doping and values. In addition, the affective states were largely diminished in the ambassadors (effect size: 0.881) according to the corresponding questionnaire items administered (pre-post evaluation, Table 1). Affective states are pivotal in public speaking as they might influence the performances of the leaders. Overall, this suggests that there is a foundation for future development through the experiences gained from leading multiple workshops with different groups that, in turn, can be used to help prepare a second generation of young ambassadors in the six project countries and beyond. Furthermore, the present study is the first to evaluate longitudinally the self-efficacy perceived by the peer educators (i.e., the ambassadors) in oral presentations for the anti-doping interventions. This study highlights the importance of training and evaluating peer educators on both their knowledge of doping and their presentation skills or other related variables (i.e., self-efficacy).

The 2020 I Run Clean follow-on project studied here was designed to build on and add to the innovative aspects of the anti-doping e-learning platform launched by European Athletics in 2017. During a period when the paradigm of the fight against doping in sport expanded from testing and punishment to include values-based education, this novel project targeted practically all groups within athletics with a unique programme comprising expanded “top-down” e-learning and newly designed “peer-to-peer” workshops delivered by young volunteer ambassadors on athletes. The scientific literature has demonstrated the effectiveness of peer education in anti-doping interventions aimed at reducing positive attitudes and moral disengagement towards doping and improving self-efficacy [41]. The recommendations for the design of anti-doping programmes included that such programmes should be peer-led and target young people and adolescents [42,43]. However, anti-doping organizations lose control over learning outcomes with peer-led approaches [16,41,44]. Educational programmes should ensure that those responsible for the programme implementation are capable of leading anti-doping workshops. In addition, most anti-doping interventions target adolescent or young athletes. A recent review conducted by Daher et al. highlighted that only two out of twenty-five screened studies were focused on coaches [41]. One key theme in the anti-doping literature was the magnitude of the role of coach–athlete relationships in the participants’ experience in sport, extending to their doping beliefs and actions. According to a recent study by Barkoukis et al., the strength and quality of the relationship between a coach and athlete is what allows the coach to influence the athlete’s beliefs, choices and behaviours, including those related to doping [38]. Values-based anti-doping education seems to have significant potential for future development, and the double-intervention approach of the I Run Clean project could cement the theoretical with personal values discussions for both athletes and coaches and eventually other groups. The current study suggests that the project has delivered important elements for realising this potential by providing positive feedback on two of the most obvious quality indicators. However, there are other research steps and aspects to be examined before final conclusions can be drawn.

A more profound understanding can be attained by examining these factors within a comprehensive framework, integrating various theoretical perspectives and exploring their interactions [42]. The interaction between the different items studied can help profile an athlete/coach and potentially enable teams to make informed decision regarding doping awareness before a *faux pas*.

The most obvious category of enquiry would be to repeat the current study’s research with more extensive samples of the second generation of ambassadors and participants from future workshops. This would be enhanced by a study with other groups participating (medical support personnel, parents, sport leaders). More extensive samples of all the target groups would facilitate comparisons between countries to determine if cultural adjustments are required.

A second category of possible research for enhancing the understanding of I Run Clean and the approach taken would be to test the impact of the e-learning aspect of the programme, the combination of the e-learning and workshop versus one or the other and the order in which a participant experiences the interventions.

A third category would be to evaluate the residual impact for the different groups (and in different countries) over a longer period, say three to six months, for single and double interventions.

## 5. Conclusions

Values-based anti-doping interventions are highly warranted to promote clean sport behaviours at the elite, grassroots and community levels. The efficacy of the tools developed in the European Athletics I Run Clean project is essential for expanding its impact and outreach. This study confirms the quality of the project design and delivery, and these confirmations can be used as leverage when engaging athletic federations outside of the project consortium and other potential partners in Europe and beyond.

## Figures and Tables

**Table 1:** Ambassadors training program effectiveness. The paired samples *t*-test show improvements both in public speaking (*p* < 0.001) and affective states (*p* < 0.001).

			Statistic	df	*p*	Mean Difference	SE Difference	Effect Size
Public Speaking PRE	Public Speaking POST	Student’s *t*	−6.75	28.0	<0.001	−0.369	0.0547	−1.254
Affective states PRE	Affective states POST	Student’s *t*	4.83	29.0	<0.001	0.467	0.0967	0.881

Note. Df = Degrees of Freedom; SE difference = Standard Error difference. Paired Samples *t*-test.

## Data Availability

The data presented in this study are aggregated and can be made available upon request from the corresponding author. The data are not publicly available due to privacy restrictions.
